# School-based physical activity and adolescent mental health: candidate psychological mechanisms, contextual moderators, and unresolved controversies

**DOI:** 10.3389/fpsyg.2026.1928334

**Published:** 2026-08-21

**Authors:** Shuting Yin, Wei Wei, Chenyang Zhao, Ling Yu

**Affiliations:** 1 Zhengdong New District Education, Culture, and Sports Bureau of Zhengzhou City, Zhengzhou, China; 2 Zhengzhou College of Finance and Economics, Zhengzhou, China; 3 Nanjing Normal University, Nanjing, China; 4 School of Sports and Health, Shanghai Lixin University of Accounting and Finance, Shanghai, China

**Keywords:** adolescents, emotion regulation, mediation, mental health, physical activity, school-based intervention, self-efficacy

## Abstract

Adolescence is a peak period for the onset of mental disorders, yet the majority of young people fail to meet recommended physical activity levels. This convergence of rising psychological distress and declining physical activity has positioned schools, settings that reach nearly all adolescents, as a central platform for population-level mental health promotion through movement. This mini review synthesizes recent evidence on the effects of school-based physical activity interventions on adolescent mental health, focusing on the psychological mechanisms that carry these effects, the contextual factors that modify them, and the controversies that remain unresolved. Evidence is kept in three tiers throughout: trials and meta-analyses of school-based interventions, which form the primary evidence base; longitudinal cohort studies; and cross-sectional or out-of-school observational studies, which are used only as supplementary evidence and cannot show how school-based interventions work. Meta-analyses and systematic reviews indicate that such interventions produce small but statistically significant improvements in depressive symptoms, anxiety, self-esteem, and social competence, although effects are inconsistent across studies, specific to particular outcomes, and are often absent in unselected samples. Mediation studies, most of them cross-sectional and few embedded in school programmes, converge on self-efficacy, self-esteem, social support, and emotion regulation as candidate pathways, frequently modeled as serial (chain) effects; such models describe statistical mediation and do not establish the mechanisms through which interventions act. Sex, age, socioeconomic status, and the school and family environment plausibly modify these effects, but moderation findings are inconsistent in direction, with stronger associations reported in girls for some outcomes and in boys for others, so recommendations for sex-specific programming remain premature. Substantial disagreement persists regarding the relative efficacy of different intervention modalities, the shape of the dose–response relationship, and whether short-term gains can be sustained, a question that itself conflates maintenance of activity behavior, persistence of psychological benefit, and long-term correlates of sport participation. We argue that methodological limitations, the neglect of social inequality, the near-absence of mediation testing within trials, and the largely untested potential of emerging delivery models constitute priority directions for a field that could yet deliver scalable and sustainable mental health benefits for young people.

## 1 Introduction

Adolescence is a period of heightened vulnerability to mental ill-health: worldwide, roughly one in seven (14%) of those aged 10–19 years experiences a mental disorder (World Health Organization, 2024). In parallel, more than 80% of adolescents fail to meet the World Health Organization's recommendation of at least 60 min of daily moderate-to-vigorous physical activity (Guthold et al., 2020). This “dual crisis,” escalating psychological distress alongside persistently low physical activity, has drawn researchers to the school, which reaches almost the entire adolescent population and offers an ideal platform for large-scale, low-cost mental health promotion (Viner et al., 2025; Ditzen-Janotta et al., 2026).

Accumulating systematic reviews and meta-analyses indicate that such interventions can improve depressive symptoms, anxiety, self-esteem, and social competence in young people (Cai et al., 2025; Fu et al., 2025; Liu et al., 2026). However, the magnitude, direction, and durability of these effects vary markedly, and some trials detect no clear benefit (da Silva et al., 2025; Ram et al., 2026). This inconsistency suggests that the relationship is not simply linear but is shaped by multiple psychological mechanisms and contextual factors. Three questions are therefore central: through which psychological pathways does physical activity affect adolescent mental health; which factors amplify or attenuate this effect; and where do competing perspectives diverge on intervention type, dose–response, and sustainability?

This mini review synthesizes current evidence on these three questions in order to inform future intervention design and policy. For each of them, we state explicitly whether the available evidence comes from school-based interventions or from observational research conducted outside them, because the two support very different conclusions.

## 2 Scope, search strategy, and evidence appraisal

This article is a narrative mini review rather than a systematic review, and the search aimed to map recent, representative evidence rather than to catalog it exhaustively. The process is described below so that readers can see how the evidence base was assembled; because screening was not systematic, we do not claim that another team would reproduce the same set of studies.

**Scope and evidence tiers**. The review is organized around school-based physical activity interventions in adolescents. Because few such interventions have tested psychological mediators or pre-specified moderators, evidence is reported in three tiers, and the tier is identified whenever a study is cited: (i) meta-analyses, systematic reviews, and randomized or cluster-randomized trials of school-based interventions (primary evidence); (ii) longitudinal cohort studies of physical activity or sport participation (evidence on temporal order); and (iii) cross-sectional surveys and studies of out-of-school or extracurricular activity (supplementary evidence, used to generate rather than to test mechanistic hypotheses). Tier iii studies are not treated as showing that an intervention works, or how it works.

**Search and eligibility**. We searched PubMed, Web of Science, Scopus, and PsycINFO for English-language records published between 1 January 2023 and 1 June 2026, combining three Boolean blocks: (“physical activity” OR exercise OR sport^*^ OR “physical education”) AND (“mental health” OR depress^*^ OR anxiety OR stress OR wellbeing OR self-esteem OR resilience OR “quality of life”) AND (adolescen^*^ OR youth OR teenager^*^ OR “high school” OR “middle school” OR school^*^). Reference lists of key articles were screened, and a few older works of continuing relevance were retained. We included studies reporting quantitative associations or effects between physical activity, exercise, or sport participation and mental health outcomes in school-age samples (mean age 10–19 years prioritized), and excluded studies of adults, of clinical populations with somatic disease, conference abstracts, and reports without extractable results. Two departures from the age criterion were deliberate and are flagged where they appear: pooled syntheses combining children with adolescents, and one quasi-experimental evaluation in 5–6-year-olds (Ram et al., 2026), discussed only as boundary evidence on universal, low-dose whole-school programmes. Records were screened by the authors against these criteria and selected for relevance to the three review questions and for methodological informativeness; screening was not duplicated independently, and no study-flow count was compiled, consistent with the narrative format.

**Appraisal**. Consistent with the narrative format, no formal risk-of-bias assessment was conducted; we instead report the certainty ratings supplied by the source syntheses where available, and state the design, sample, exposure, and outcome of each primary study. This limitation is revisited in Section 8.

**Outcome taxonomy**. “Mental health” covers heterogeneous constructs in this literature, so we distinguish (a) psychopathological symptoms (depressive and anxiety symptoms, perceived stress, internalizing and externalizing problems, non-suicidal self-injury); (b) positive mental health (self-esteem, resilience, life satisfaction, subjective wellbeing, social competence, health-related quality of life); and (c) related but distinct constructs (sleep, cognition, academic achievement, antisocial behavior), treated as candidate mediators or as separate outcomes rather than as mental health outcomes in themselves. Estimates are reported with the construct and metric to which they refer.

## 3 Overall effects of school-based physical activity interventions on adolescent mental health

Several large-scale meta-analyses and systematic reviews provide a reasonably consistent basis for the overall mental health effects of school-based physical activity interventions. The pooled estimates below indicate order of magnitude only: these syntheses differ in population, comparator, instrument, and sign convention, and are not directly comparable. In an umbrella review of 21 meta-analyses in children and adolescents, Cai et al. (2025) reported weak but significant effects across physical and psychological indicators, including a standardized mean difference (SMD) of −0.21 for depressive symptoms and 0.40 for cognition (a cognitive, not a mental health, outcome here). A meta-analysis of 30 randomized controlled trials by Fu et al. (2025) reported a pooled SMD of 0.37 aggregated across the heterogeneous outcomes those authors classified as mental health, with the strongest effects on stress reduction (SMD = 0.86) and social competence (SMD = 0.56); combining symptom with positive mental health measures, this aggregate is an average across outcome families rather than an effect on a single construct.

Effects differ across domains. In 17 studies of health-related quality of life, Bermejo-Cantarero et al. (2024) found small improvements in overall quality of life (ES = 0.179) and psychological wellbeing (ES = 0.158). Using the RE-AIM framework, Li et al. (2026) reported improved perceived fitness (SMD = 0.31) and lower anxiety, depression, and stress (MD = −0.53); both syntheses pool children with adolescents.

Benefits are not uniformly supported. In a systematic review of 25 randomized controlled trials, Ditzen-Janotta et al. (2026) found positive effects in only 12 studies, with meta-analysis showing a significant improvement solely for health-related quality of life (SMD = 1.08); depression, anxiety, and psychological difficulties were non-significant. Similarly, a cluster randomized controlled trial in Brazilian adolescents by da Silva et al. (2025) found no improvement in the full sample, with reductions in depression (MD = −0.97) and anxiety (MD = −3.03) only in the subgroup with moderate-to-severe baseline symptoms. Baseline psychological risk may therefore condition the size of the effect, consistent with Liu et al. (2026), whose effect in high-risk or trait subgroups (SMD = 0.31) far exceeded that in the general population (SMD = 0.16). Both estimates come from subgroup contrasts rather than from formal tests of interaction, however, so this is a possibility awaiting confirmation in trials designed to compare risk strata, not an established moderator.

Evidence on persistence is easily over-read. In a meta-analysis of 90 school-based intervention studies, Moin et al. (2026) found that interventions increased physical activity in the short term (0–6 months; SMD = 0.41) but not at follow-ups beyond 6 months; the outcome pooled in that synthesis is activity behavior rather than mental health, so the finding shows that behavior change is poorly maintained, not that psychological benefits disappear. A systematic review by Viskari et al. (2025) reported mixed effects on internalizing and externalizing symptoms and psychological wellbeing, with positive effects only for social wellbeing.

## 4 Candidate psychological mechanisms: mediating and serial-mediating pathways

Physical activity is widely held to affect mental health through mediating and serial-mediating pathways, and identifying them would sharpen intervention targets. Two caveats govern this section. First, mediation tested within a trial, where the intervention is shown to change the mediator and that change to carry the effect on the outcome, answers a different question from mediation estimated in cross-sectional data, where exposure, mediator, and outcome are measured simultaneously and temporal order is assumed rather than observed. Second, “full mediation” in such models describes the fit of a specified model, not a causal pathway. Almost all the evidence below is of the second kind, and the constructs it implicates are therefore treated as candidate mechanisms.

### 4.1 Mediators tested within intervention trials

This is the smallest body of evidence, and it is largely negative. In the Burn 2 Learn trial, Leahy et al. (2023) found that sleep-related variables did not mediate intervention effects on psychological stress and internalizing problems; it is the only school-based trial identified in this review that has tested a mediator of a mental health outcome. The Movimente cluster randomized controlled trial concerns a different outcome and is cited here for that contrast: Aragoni da Silva et al. (2024) tested psychosocial mediators of the intervention's effect on physical activity, not on mental health. The intervention improved perceptions of the school environment and teacher support, but neither mediated the change in activity. The trial shows how rarely psychosocial pathways are confirmed even for behavioral outcomes, and should not be read as evidence about mental health mechanisms. We identified no school-based trial confirming self-efficacy, self-esteem, or emotion regulation as a mediator of mental health effects; this absence, rather than any positive finding, characterizes the mechanistic evidence base in schools.

### 4.2 Longitudinal cohort evidence

In a longitudinal analysis of 4,216 children in the Generation R cohort, Rodriguez-Ayllon et al. (2023) found that self-esteem significantly mediated the association between sport participation and internalizing symptoms (indirect effect β = −0.009), whereas neurobiological and behavioral variables did not. The design supports temporal ordering, but the exposure is participation in sport rather than an intervention, and the indirect effect is very small.

### 4.3 Cross-sectional mediation models (supplementary evidence)

The four constructs most often proposed as mechanisms rest almost entirely on this tier. Self-efficacy is the most widely studied. In 400 Chinese secondary school students, Zhang et al. (2024) found the physical activity–mental health relationship statistically fully mediated by self-efficacy and stress self-management in a chain-mediating pathway. In 1,732 high school students, Li et al. (2024b) found physical activity predicted resilience both directly and indirectly, through self-efficacy and basic psychological needs.

Self-esteem is a second candidate pathway. In 484 high school students, Li and Hao (2025) reported chain mediation through social support and self-esteem for life satisfaction and depressive symptoms; these serial indirect effects were statistically significant but very small, and lacked the confidence intervals, scaling, and proportion of total effect needed to judge their practical importance, so the coefficients are not reproduced here. Feng et al. (2024) similarly reported self-esteem and social anxiety as statistical full mediators in a post-COVID-19 sample.

The remaining studies in this tier address different outcomes, and their pathways are not interchangeable. A survey of 7,272 adolescents by Wang et al. (2025) found that physical activity suppressed antisocial behavior through the chain mediation of positive peer relationships and subjective wellbeing (serial mediating effect 16.49%)—a behavioral, not a symptom, outcome. Using PISA data from 113,203 students, Li et al. (2024a) found emotion regulation mediated the association between extracurricular sport participation and subjective wellbeing. In 1,579 students, Yao et al. (2025) reported serial mediation by meaning in life and resilience between physical activity and sleep quality (largest path, 34.48%).

These studies make self-efficacy, self-esteem, social support, and emotion regulation plausible candidates, but cannot show that school-based interventions operate through them, and the negative trial analyses above caution against assuming that they do. Mediation analyses pre-specified within trials, with mediators measured before outcomes, are the missing step.

## 5 Contextual moderators and correlates of participation

Three kinds of finding are commonly reported together as moderation and are separated here: formally tested moderation (an interaction term in a trial, or a subgroup contrast in meta-regression); differences in the strength of association between subgroups in observational data; and correlates of participation, which predict how much activity adolescents undertake but not whether they modify an intervention's mental health effect.

Sex is the most thoroughly studied moderator, and the findings do not converge. In 26,974 Canadian adolescents, Oberle et al. (2025) found meeting 24-h movement guidelines more strongly associated with mental wellbeing among girls (a cross-sectional survey whose exposure is guideline adherence, not a school programme). In 2,270 Shanghai students, however, Bai et al. (2026) found stronger associations with perceived stress, resilience, and self-efficacy in boys. In meta-analytic subgroup analyses, Fu et al. (2025) reported larger pooled effects in boys (SMD = 1.11), whereas Hua et al. (2025), in 15,344 adolescents, found the protective association stronger in girls and younger adolescents. Wang et al. (2025) found the suppressive effect on antisocial behavior more pronounced in boys. The direction of sex differences therefore varies with the outcome, the design, and the exposure, and most estimates come from subgroup analyses that were neither pre-specified nor powered to detect interaction. On present evidence, sex-specific programme design is not warranted; pre-specified, adequately powered moderation testing with sex-disaggregated reporting is.

Age is likewise important, though here too the evidence is largely associational. Fu et al. (2025) reported larger effects for middle-school students (SMD = 0.45). In a longitudinal Japanese study, Kawai and Yamagata (2024) found elevated risk of depressive tendencies among adolescents shifting from active in fifth grade to inactive in eighth grade, indicating that the trajectory of activity predicts mental health better than a single time point.

The role of socioeconomic status is increasingly recognized. In 1,285 Swedish adolescents, Dahlstrand et al. (2025) found a gradient between socioeconomic status and mental health problems that was partially mediated by out-of-school moderate-to-vigorous physical activity; among girls, vigorous activity mediated 6.8%−9.2% of these associations (a mediation rather than a moderation finding). In a systematic review of 44 school interventions, Haataja et al. (2025) found differential-effect analyses across sociodemographic subgroups very limited and mostly non-significant.

School and family environments shape participation, and may or may not shape the psychological consequences of participating. In 4,617 Thai adolescents, Amornsriwatanakul et al. (2023) found facilities and home equipment associated with participation, which was also higher where parents exercised weekly (OR = 1.36) or gave three or more support types (OR = 1.43); the outcome of that cross-sectional survey is participation itself, so these are correlates of engagement rather than demonstrated moderators of mental health effects, cited to inform implementation rather than to explain outcomes. A New Zealand modeling study by Bergen et al. (2024) estimated the largest gains in total activity from natural-environment play areas and in physical literacy from peer-led coaching, both activity rather than mental health outcomes. In a longitudinal cohort, Ramer et al. (2025) found childhood enjoyment of activity predicted fitness participation at age 26, mediated by high school sport—an observational pathway spanning two decades, not an intervention effect.

## 6 Controversies and competing perspectives: intervention type, dose–response, and sustainability

On modality, Liu et al. (2026) found that intervention type significantly moderated mental health effects (χ^2^ = 8.42, *p* = 0.04): mind-body interventions yielded the largest effect (SMD = 0.32), with multicomponent or behavioral interventions next (SMD = 0.18) and sport/games and aerobic training non-significant. These are between-study comparisons rather than head-to-head trials, so modality remains confounded with population, outcome, and dose. A narrative review by Li et al. (2024c) similarly linked different modalities to different psychological targets. In 63 adolescent athletes and non-athletes, Pasquerella et al. (2025) found that strategic sports reduced stress whereas self-paced exercise enhanced cognitive flexibility—a small comparison of athletes and non-athletes outside any school programme, and therefore hypothesis-generating.

The dose–response relationship is also contested. Bai et al. (2026) found a non-linear cross-sectional association: gains in perceived stress, resilience, and self-efficacy were steepest at low activity levels and attenuated markedly at high levels, supporting a “minimum effective dose” hypothesis whereby modest increases may benefit sedentary adolescents; a curve estimated from between-person differences need not, however, describe what follows when an individual's activity is increased. For depressed adolescents, Yan et al. (2025) proposed more than 3 weekly sessions of under 60 min sustained beyond 8 weeks, an inference from clinical samples that may not transfer to universal programmes. Yet Smith et al. (2025) found no differential effect of light vs. moderate-to-vigorous activity on cortisol stress reactivity, although both groups improved on self-reported mental health (*d* = −0.38 to −0.54).

Disagreement over sustainability is most pronounced, but part of it is definitional. Three distinct questions are involved: whether behavior change is maintained, whether psychological benefits persist once an intervention ends, and whether adolescent sport participation predicts adult outcomes. Moin et al. (2026) address the first: pooled effects on activity were significant in the short term (SMD = 0.41) and were not maintained beyond 6 months. The second is the least well answered, because few trials follow psychological outcomes beyond the intervention period; Viskari et al. (2025) found interventions using environmental modification and external delivery agents outperformed those run by school staff without a protocol, which bears on implementation rather than durability. The third is addressed by Ramer et al. (2025), who found that high school sport participation still predicted wellbeing and depression at age 26, an observational association rather than evidence that an intervention effect persists. None of these findings addresses why effects endure or fade. That programmes cultivating enjoyment, intrinsic motivation, and self-regulation yield more durable outcomes than those which raise short-term activity alone is a hypothesis worth testing rather than a conclusion supported by the evidence reviewed here; no trial in this literature has compared programmes on that basis.

Controversy extends to specific outcomes. In a 12-month cluster randomized controlled trial, Werneck et al. (2026) found no effect of aerobic exercise on non-suicidal self-injury in the full sample, with a 49% reduction in incidence (IRR = 0.49) only among participants with low baseline physical activity (again a subgroup finding). Although some studies support benefits for cognition and academic achievement (Marsigliante et al., 2023; Latino et al., 2023), a quasi-experimental study of the Daily Mile by Ram et al. (2026) found no effect on physical activity, mental health, or academic performance in 5–6-year-olds. That sample lies below the adolescent range and is retained only because it evaluates a universal, low-dose, whole-school design also used in secondary schools; the null result is instructive for, but not generalizable to, adolescents.

## 7 Research gaps and future directions

Methodological limitations are the foremost barrier to stronger evidence. In a systematic review of 148 studies of 24-h movement guidelines, Hossian et al. (2025) found that 87% were cross-sectional and only 3% of observational studies were rated high quality. Ditzen-Janotta et al. (2026) rated certainty as “very low” for most outcomes, and Wendel et al. (2024) found almost all COVID-19-related intervention studies at high risk of bias. Small samples, weak randomization, absent blinding, short follow-up, and non-standardized outcomes call for more rigorous designs and validated instruments. Reporting practice compounds this: indirect effects are frequently declared significant without confidence intervals, scale, or proportion of the total effect, so statistical significance is reported where psychological significance cannot be judged.

Social inequality has long been neglected. Beyond the limited subgroup analyses noted by Haataja et al. (2025), Oyeyemi et al. (2026) emphasized that adolescents in low- and middle-income countries remain severely understudied, and Winsper et al. (2026) found that school programs there performed better on proximal than on distal outcomes, with cultural adaptation and implementation quality decisive. Future work should explicitly test whether socioeconomic status, ethnicity, and migration background moderate effects.

Emerging approaches offer new opportunities. Kang et al. (2024) highlighted digital technology and artificial intelligence for augmenting team-sport benefits; Andermo et al. (2026) proposed combining physical activity with homework support; and a case study by St Pierre et al. (2024) illustrated a “near-peer coach” model, though a single case study cannot establish effectiveness. A systematic review of peer-led behavioral interventions by Brinsley et al. (2025) found evidence still insufficient to support significant mental health effects on any outcome, indicating that emerging models require more rigorous evaluation.

Integrating 24-h movement behaviors represents a further paradigm: adherence is low worldwide (Hossian et al., 2025), protective thresholds for sleep, exercise, and screen time have been identified in 33,397 Chinese adolescents (Qu et al., 2025), and meeting even one recommendation is associated with better mental wellbeing (Oberle et al., 2025). These findings argue for shifting from physical activity alone toward integrated optimization of 24-h movement behavior, although they are cross-sectional and describe the context in which school programmes operate rather than their effects (Table 1).

**Table 1:** Psychological pathways, contextual moderators, and directions for the design and evaluation of school-based physical activity interventions for adolescent mental health.

Evidence domain	Synthesis of current evidence	Evidence base (design and tier)	Representative findings and references	Directions for programme design and evaluation
Overall effect profile	Evidence supports small but significant benefits for depressive symptoms, anxiety, self-esteem, social competence, and quality of life; however, null findings and outcome-specific effects remain common, and estimates from different syntheses are not directly comparable.	Umbrella reviews, meta-analyses of RCTs, individual cluster RCTs (Tier i)	Umbrella reviews and meta-analyses report weak-to-moderate effects (Cai et al., 2025; Fu et al., 2025; Bermejo-Cantarero et al., 2024; Li et al., 2026), whereas several RCTs show benefit only for selected outcomes or symptomatic subgroups (Ditzen-Janotta et al., 2026; da Silva et al., 2025; Viskari et al., 2025).	Define the primary psychological target in advance and avoid framing physical activity as a uniformly effective mental health intervention.
Baseline risk	Effects may be stronger and more consistent among adolescents with elevated symptoms or higher-risk traits than in unselected school populations, but this rests on subgroup contrasts rather than formal tests of interaction and awaits confirmation.	*Post hoc* subgroup analyses within RCTs and meta-analyses (Tier i); no formal interaction tests	High-risk or moderate-to-severe baseline symptom groups show larger improvements than general samples (Liu et al., 2026; da Silva et al., 2025; Werneck et al., 2026).	Test the possibility directly: pre-specify stratification by baseline symptoms and report formal interaction tests before targeting components by risk.
Self-efficacy and basic psychological needs	Self-efficacy is the most frequently proposed candidate mediator, but the supporting models are almost entirely cross-sectional and were not estimated within school interventions; serial pathways with stress self-management or need satisfaction remain hypotheses.	Cross-sectional mediation models (Tier iii); no confirmatory trial evidence	Chain mediation links physical activity to mental health or resilience through self-efficacy, stress self-management, and need satisfaction (Zhang et al., 2024; Li et al., 2024b).	Mastery experiences, graded goals, competence feedback, and autonomy support are plausible components to evaluate rather than components with demonstrated mediation in schools; measure the proposed mediator prospectively so the pathway can be tested.
Self-esteem, social support, and peer relations	Social support, self-esteem, peer relationships, and belonging are recurrent psychosocial pathways connecting physical activity with better well-being and fewer internalizing or maladaptive outcomes, though one longitudinal cohort supports only self-esteem, with a very small indirect effect.	One longitudinal cohort (Tier ii); otherwise cross-sectional (Tier iii)	Serial mediation through social support and self-esteem (Li and Hao, 2025; Feng et al., 2024), and through peer relationships and subjective wellbeing for antisocial behavior (Wang et al., 2025); self-esteem in the Generation R cohort (Rodriguez-Ayllon et al., 2023).	Cooperative, socially safe, and inclusive formats are worth evaluating on this rationale, which is theoretical rather than demonstrated in school trials; avoid designs that intensify comparison or exclusion among less-skilled students.
Emotion regulation, resilience, and sleep	Emotion regulation and resilience are plausible candidate mediators supported only by survey data, whereas sleep was tested directly within a trial and did not mediate.	Cross-sectional and large-scale survey models (Tier iii); one RCT mediation analysis (Tier i)	Survey studies support emotion-regulation and resilience pathways (Li et al., 2024a; Yao et al., 2025); sleep did not mediate in the Burn 2 Learn trial (Leahy et al., 2023).	Coping-skills practice, reflection, and recovery education are candidate components for evaluation; treat psychosocial pathways as hypotheses to be tested rather than as established mechanisms.
Sex, age, and socioeconomic inequality	Sex differences are inconsistent in direction and depend on the outcome examined; age effects are largely associational; socioeconomic moderation is essentially untested, although physical activity partly mediates mental health gradients.	Non-pre-specified subgroup contrasts in surveys and meta-analyses; one systematic review of differential effects	Stronger associations in girls for some outcomes (Oberle et al., 2025; Hua et al., 2025) and in boys for others (Fu et al., 2025; Wang et al., 2025; Bai et al., 2026); subgroup analyses by socioeconomic status are limited and mostly non-significant (Haataja et al., 2025).	Pre-specify and power moderation analyses and report sex-disaggregated results; on current evidence, do not design sex-specific programmes. Design low-cost, equitable programs that address access, equipment, time, and safe participation.
School and family ecology	Teacher support, school climate, facilities, home equipment, parental support, and community resources are established correlates of participation; their role as moderators of mental health effects has not been demonstrated.	Cross-sectional population survey; modeling study; one trial mediation analysis	Facilities, home equipment, and parental support predict participation (Amornsriwatanakul et al., 2023); modeled effects concern activity and physical literacy (Bergen et al., 2024); improved school-environment perceptions did not mediate the intervention's effect on physical activity (Aragoni da Silva et al., 2024).	Whole-school approaches combining teacher engagement, supportive spaces, family communication, and community-linked opportunities are a reasonable avenue to trial, but the evidence links these factors to participation rather than to mental health; test whether they modify psychological outcomes.
Intervention modality and dose	No single modality is clearly superior across outcomes; modalities may act on different psychological mechanisms and dose–response may be non-linear, but comparisons are between studies rather than head-to-head, and dose–response curves are estimated from between-person differences.	Meta-regression and subgroup comparison; cross-sectional dose–response modeling; one 3-arm RCT	Larger pooled effects for mind-body or multicomponent approaches (Liu et al., 2026; Li et al., 2024c); steepest gains at low activity levels (Bai et al., 2026); no differential effect of intensity on cortisol reactivity (Smith et al., 2025).	Match modality to target mechanism and report FITT details; test pragmatic doses that schools can implement and sustain.
Sustainability and transfer	Three questions are commonly conflated: maintenance of activity behavior, persistence of psychological benefit after an intervention ends, and long-term correlates of adolescent sport participation. Only the first has been assessed meta-analytically; the second is largely untested.	Meta-analysis with activity outcomes; systematic review; one longitudinal cohort	Activity gains attenuate beyond 6 months (Moin et al., 2026); psychological effects are mixed (Viskari et al., 2025); adolescent sport participation predicts wellbeing at age 26 (Ramer et al., 2025).	Extend follow-up of psychological outcomes specifically and measure habit formation, enjoyment, motivation, and post-intervention participation opportunities, so that the durability hypothesis can be tested rather than assumed.
Emerging models and evidence gaps	Digital tools, artificial intelligence, near-peer coaching, and 24-h movement frameworks are promising but require stronger causal and equity-focused evaluation.	Narrative reviews, a trial protocol, a case study, and one meta-analysis of peer-led interventions	Digital and AI augmentation, near-peer delivery, and integrated activity–sleep–sedentary models (Hossian et al., 2025; Kang et al., 2024; Andermo et al., 2026; St Pierre et al., 2024); peer-led evidence remains non-significant (Brinsley et al., 2025).	Use longitudinal, adequately powered, mechanism-informed, and equity-sensitive trials, especially in low- and middle-income settings.

Numbers in parentheses refer to the reference list. The final column sets out directions for design and evaluation, not components supported by mediation evidence in school-based interventions. Tier i = meta-analyses, systematic reviews, and randomized or cluster-randomized trials of school-based interventions; Tier ii = longitudinal cohort studies; Tier iii = cross-sectional or out-of-school observational studies (see Section 2). FITT, frequency, intensity, time, and type; PA, physical activity; RCT, randomized controlled trial.

## 8 Discussion

Current evidence indicates that school-based physical activity interventions have small, outcome-specific benefits for adolescent mental health, clearest for health-related quality of life, perceived stress, and social competence, and for depressive and anxiety symptoms among adolescents who are already symptomatic; in unselected school populations null results are common. Self-efficacy, self-esteem, social support, and emotion regulation are the leading candidate mediators, but they have been supported mainly in cross-sectional models estimated outside intervention contexts and remain untested within school trials, while sex, age, socioeconomic status, and the school environment are plausible moderators whose reported effects are at present inconsistent in direction. Where tailoring is considered, baseline psychological risk is the more promising candidate, since the pattern is at least consistent in direction, but it too rests on subgroup contrasts and should be tested before it is acted upon; sex, for which the direction of findings is inconsistent, is not a defensible basis for tailoring at present.

This review has limitations of its own. It is narrative rather than systematic; the search was restricted to English-language publications and concentrated on 2023–2026, so earlier foundational trials and non-English evidence are under-represented; no formal risk-of-bias or certainty appraisal was undertaken; and a substantial part of the mechanistic and contextual evidence is cross-sectional and drawn from settings other than school programmes. The synthesis should be read as a map of a contested field rather than as a pooled estimate of effect.

No consensus has emerged on differential effects of intervention type, dose–response, or sustainability; social inequality has been persistently overlooked; and research from low- and middle-income countries remains scarce.

Future research should prioritize longitudinal and experimental designs; test candidate mediators prospectively within trials, with mediators measured before outcomes and analyses pre-specified; pre-specify and adequately power moderation analyses and report results disaggregated by sex and socioeconomic status; report effect sizes with confidence intervals and, where possible, indices of clinically meaningful change; extend follow-up, distinguishing maintenance of activity from persistence of psychological benefit; integrate the 24-h movement framework; and strengthen research in low- and middle-income countries. Through these efforts, school-based physical activity interventions could become an effective, scalable, and sustainable public health strategy for promoting adolescent mental health.
